# Magnetic Resonance Imaging Findings of Term and Preterm Hypoxic-Ischemic Encephalopathy: A Review of Relevant Animal Models and Correlation to Human Imaging

**DOI:** 10.2174/1874440001812010055

**Published:** 2018-10-17

**Authors:** Kyle A. Jisa, Dillon D. Clarey, Eric S. Peeples

**Affiliations:** Department of Pediatrics, University of Nebraska Medical Center, Omaha, NE, United States

**Keywords:** Asphyxia, Neonate, Brain injury, Mouse, Rat, Rabbit, Sheep, Non-human primate

## Abstract

**Background:**

Neonatal hypoxic-ischemic encephalopathy is brain injury caused by decreased perfusion and oxygen delivery that most commonly occurs in the context of delivery complications such as umbilical cord compression or placental abruption. Imaging is a key component for guiding treatment and prediction of prognosis, and the most sensitive clinical imaging modality for the brain injury patterns seen in hypoxic-ischemic encephalopathy is magnetic resonance imaging.

**Objective:**

The goal of this review is to compare magnetic resonance imaging findings demonstrated in the available animal models of hypoxic-ischemic encephalopathy to those found in preterm (≤ 36 weeks) and term (>36 weeks) human neonates with hypoxic-ischemic encephalopathy, with special attention to the strengths and weaknesses of each model.

**Methods:**

A structured literature search was performed independently by two authors and the results of the searches were compiled. Animal model, human brain age equivalency, mechanism of injury, and area of brain injury were recorded for comparison to imaging findings in preterm and term human neonates with hypoxic-ischemic encephalopathy.

**Conclusion:**

Numerous animal models have been developed to better elicit the expected findings that occur after HIE by allowing investigators to control many of the clinical variables that result in injury. Although modeling the same disease process, magnetic resonance imaging findings in the animal models vary with the species and methods used to induce hypoxia and ischemia. The further development of animal models of HIE should include a focus on comparing imaging findings, and not just pathologic findings, to human studies.

## 1 INTRODUCTION

Neonatal Hypoxic-Ischemic Encephalopathy (HIE) is a condition resulting from decreased blood flow and oxygen delivery to the brain, which generally occurs at or around the time of delivery. Despite the current standard treatment with therapeutic hypothermia, many infants suffering from HIE ultimately die or develop cerebral palsy, epilepsy, or other neurodevelopmental impairment [1, 2]. Various animal models have been developed to examine changes within the brain during Hypoxic-Ischemic (HI) injury, as well as to test novel therapies. Use of these models allows for controlling multiple variables that can impact the severity of HI injury, including developmental maturity at the time of injury, duration and depth of hypoxia, location of the insult within the brain, and single *versus* repetitive HI insults [3]. The ability to alter these variables within animal models has better elicited how the neonatal brain is affected by and recovers from HI injury.

Clinically, one of the exams that can help to define the severity of the HI injury on the neonatal brain is Magnetic Resonance Imaging (MRI). MRI has been identified to have the highest sensitivity and specificity for clinically evaluating changes in myelin in a non-invasive manner [4]. Due to the lack of significantly associated radiation and its non-invasiveness, substantial clinical research has been performed to describe the characteristic patterns seen on MRI after neonatal HIE. Various animal models have been utilized for inducing cerebral ischemia, but there have only been a few studies that have focused on imaging these animals with HIE [2, 4-10]. Translating these studies to human populations is difficult due to the need to adjust for different rates of brain maturation between species, complicated by the fact that MRI findings in animals vary based on the species and method of inducing hypoxia and ischemia.

This review of the current literature regarding MRI findings in animal models of HIE will aid investigators in evaluating the available models to select the most appropriate species and mechanism of interest to fit the process that they are studying. This manuscript will review the typical MRI findings in preterm (≤36 weeks) and term (>36 weeks) neonates affected by HIE, as well as the animal models that have been utilized for modeling HIE. Additionally, it will compare the typical MRI findings in human and animal models to better delineate shared patterns. Pathological findings are included when necessary due to the paucity of pre-clinical imaging data in some animal models.

## 2 METHODS

### 2.1 Types of Studies

Included studies were restricted to *in vivo* animal studies with induced HI injury to animal models and at least one primary MRI finding reported. All methods of HI induction were included. Only studies with English language abstracts were eligible for inclusion.

### 2.2 Types of Specimens

All animal models with a known brain age equivalency to a preterm or term human neonate were eligible for inclusion.

### 2.3 Types of Outcome Measures

Study data that were recorded included species of animal model, age of the animal model at the time of HI injury, brain-age equivalency to human neonates, method used to induce HI injury, alteration in oxygen level exposure, time length for inducing HI injury, the timing of when MRI was performed, and the findings seen on brain imaging.

### 2.4 Search Strategy

Literature databases including MEDLINE, EMBASE, and Cochrane Library were searched by two authors (KJ, DC) independently. Databases were searched using “hypoxic-ischemic encephalopathy,” “HIE,” or “neonatal encephalopathy” in combination with “magnetic resonance imaging” or “MRI.” All manuscript titles of the database searches were critically reviewed. If ambiguity existed over the relevance of a manuscript’s title to this research, the abstract was reviewed for relevance. Abstracts of all manuscript titles deemed initially relevant to the scope of this paper were reviewed for applicability. If ambiguity existed after abstract review, a full review of the manuscript was performed to determine if the paper was pertinent. A full review of references was manually performed on all manuscripts that were deemed by both reviewing authors to be relevant to the scope of this research. Additionally, the references from each included manuscript were reviewed for additional relevant articles.

## 3 DISCUSSION

### 3.1 Magnetic Resonance Imaging Background

A pre-requisite to understanding descriptions and comparisons of MR imaging findings is to first understand the most common sequences that are utilized in imaging HIE. These sequences include T1-weighted, T2-weighted, Diffusion Weighted Imaging (DWI) and the resulting Apparent Diffusion Coefficient (ADC). MRI is, at its core, a visual interpretation of the changes in protons within the brain after excitation with electromagnetic radiation. T1-weighted imaging is produced from the longitudinal component of the energy produced by relaxation of the protons, whereas T2-weighted imaging results from the transverse relaxation energy. On imaging, fluids such as cerebral spinal fluid or interstitial edema will result in low signal (*i.e.* dark areas) on T1-weighted imaging and high signal (*i.e.* bright areas) on T2-weighted imaging [11]. A third magnetic resonance sequence, termed DWI, relies on the movements of protons within water, allowing for measurement of intracellular edema. To quantify the impedance of protons within water, the DWI sequence produces a value termed ADC.

In animal models, several technical aspects of magnetic resonance imaging must be considered, including field strength. Although the details of these concerns are outside the scope of this particular paper, we would refer the reader to the review by Hoyer *et al.* if they are interested in greater detail [12]. In general, animal imaging typically requires the combination of higher field powers and gradient systems that may not be typically utilized in human scanners. These modifications allow for higher resolution necessary to image the smaller animal brain. Additionally, placement of radiofrequency coils while imaging animal brains assists in significantly improving the signal to noise ratio as well as the image quality.

### 3.2 Magnetic Resonance Imaging of Preterm Neonates

Few studies have assessed early imaging patterns in severe hypoxia-ischemia in preterm neonates, due to the wide spectrum of HI injury in preterm infants given its relation to frequent intermittent hypoxic events as opposed to a single sentinel event. The few studies of MRI findings in this population have demonstrated that after severe asphyxia, the primary injury occurs within the white matter and basal ganglia, with injury to the brainstem being less common [13-15]. This is primarily thought to be due to the redistribution of blood flow from the anterior to the posterior cerebral circulation. Of note, the pattern of injury continues to evolve through maturation. For example, severe HI in very preterm infants <32 weeks gestation results in decreased basal ganglia injury compared to later gestation, which could be due to the onset of myelination in the basal ganglia between 33 and 35 weeks gestation resulting in increased metabolic demand of that area [14].

T1- and T2-weighted imaging generally show no abnormalities on the first day after HI injury [16]. Within 2 days after injury, hyperintense signals develop on T2-weighted imaging within the thalami [16]. By day 3, T1-weighted imaging demonstrates hyperintense signal in injured regions such as the globus pallidus and Ventrolateral (VL) thalami, with decreased signal in the Posterior Limb of Internal Capsule (PLIC). Involvement of the hippocampus appears to be rare in preterm neonates with HI [16]. DWI generally demonstrates decreased diffusion within the first 24 hours, and by the third to fifth day there is decreased diffusion in the thalami that will pseudonormalize over the following week [17]. MR spectroscopy of preterm neonates during the acute phase after HI injury generally demonstrates a metabolic profile of increased lactate, myoinositol, and decreased NAA [18-20]. In the chronic stage of injury, MR imaging shows calcification and shrinkage of the thalami and basal ganglia as well as a decrease in the overall volume of the white matter [14, 21-24]. It has been hypothesized that the decrease in the cerebral white matter is secondary to loss of axons in the thalamocortical, corticothalamic, and corticoputaminal pathways [14]. This is problematic as damage to these pathways can have an impact on sensory and injury responsiveness [25].

#### 3.2.1 Murine Model

To model preterm HI brain injury, various strains of rats and mice have been utilized, with ages ranging up to postnatal day 7. Postnatal day 7 correlates with 33 weeks gestation of human brain growth in the mouse and 34 weeks in the rat [1]. As postnatal day 2 in both mice and rats corresponds to approximately 22 weeks gestation in the human neonate, any injury induced prior to that would be more consistent with fetal injury. The majority of the studies induced injury at 7 days postnatal age while other studies utilized fetal, postnatal day 3, and postnatal day 5 animals for HI injury. Methods used to induce hypoxic-ischemic injury on the murine brain include unilateral Common Carotid Artery Occlusion (CCAO), bilateral CCAO, and Middle Cerebral Artery Occlusion (MCAO). The animals are then exposed to 6.5 - 10% oxygen (with 8% the most common) for a period ranging from 10 minutes to 3.5 hours depending on species and strain [26-30].

Published MRI studies of murine models have been performed between birth and 11 weeks of life, with the earliest imaging on the day of birth demonstrating increased ADC in the cingulum and lateral external capsule [26, 31-38]. MRI of the same animals on postnatal day 7 demonstrated a decreased ADC in the cingulum and external capsule, corresponding to significant gliosis seen on pathology at postnatal day 14 [31]. The involvement of the external capsule white matter in this model is notably different from the preterm human infants who do not typically display basal ganglia injury. Conversely, the involvement of the cingulate cortex of the limbic lobe after HI injury to the fetal rat brain is similar to preterm neonates who most commonly demonstrate injury to the white matter after HI injury.

Mice with HI injury at postnatal day 3 have demonstrated ipsilateral ventriculomegaly after unilateral CCAO along with brain atrophy or porencephalic cyst formation and injury to the sensorimotor cortex, striatum, and hippocampus when MRI and subsequent pathological analysis was performed at 10 weeks after the insult [26]. Similarities to preterm neonates are displayed by the involvement of the cortical white matter which is commonly seen. However, these findings differ from those of human preterm neonates, where injury is not frequently demonstrated in the striatum of the basal ganglia or the hippocampus in very early prematurity.

Rats with HI injury at postnatal day 5 *via* bilateral CCAO demonstrated damage predominantly in the internal capsule and rare injury to the cerebral cortex which was visualized with only pathological analysis [30]. Involvement of the internal capsule in postnatal day 5 mice is anticipated to occur in a late preterm neonate secondary to the basal ganglia areas becoming more metabolically active as the neonate becomes closer to term gestation. Additionally, the involvement of the cortex in postnatal day 5 mice is in line with previously discussed postnatal day 3 murine models that also display cortical involvement after HI injury and demonstration of increasing cortical involvement would be expected in equivalent preterm neonates.

Rats and mice that underwent HI injury at postnatal day 7 have displayed multiple injured areas after both unilateral and bilateral CCAO. Areas that have been visualized to experience damage include the white matter, Periventricular White Matter (PVWM), corpus callosum, cortex, subcortex, striatum (caudate and putamen), hippocampus, and thalamus [27-29, 31-39]. Follow-up imaging has also displayed brain atrophy [33]. Notably, cortical involvement in postnatal day 7 mice begins to better coincide with the areas affected in late preterm neonates because the brain age equivalency approaches 36 weeks gestation. However, hippocampal involvement seen in mice at 7 days postnatal with MRI and neuropathology is not typically expected to occur in preterm neonates with HIE. Both preterm human and equivalent murine models should be expected to display signs of brain atrophy at long-term follow-up after HI injury.

Clear replication of HIE in the preterm neonate by murine models has been performed with varying degrees of success. Notably, the involvement of the basal ganglia has been consistently demonstrated across fetal to postnatal day 7 murine models which correlates with the injury seen in the late preterm neonate, but is inconsistent with the relative sparing of the basal ganglia seen in very preterm human infants. White matter injury to the corpus callosum and cingulate cortex seen in the murine model can be expected in the preterm neonate. Additionally, hippocampal injury seen in the murine models is not readily expected to occur in the preterm neonate which investigators should be aware of when translating these data into preterm neonates. The postnatal day 7 rat model does appear to be the best model for representing overall damage of the late preterm neonate by involving commonly injured areas such as the white matter, basal ganglia, and thalamus and because it is the most well-studied. Future research should assess the utility and accuracy of imaging less mature murine models due to the paucity of research and the growing viability of neonates at the early extremes of prematurity.

#### 3.2.2 Rabbit Model

Rabbits have also been utilized as a model for neonatal HIE, with injury induced at 21 to 29 days gestation. Fetal rabbits at 22 days gestation are equivalent to a 22 to 27 week gestational age human [40]. Rabbit fetuses exposed to HI injury through uterine artery occlusion have been imaged by MRI at postnatal day 1 and 5. Injury was primarily demonstrated in the white matter, including the corpus callosum, internal capsule, corona radiata, and the anterior commissure. Notably, only the clinically hypertonic rabbits were found to have white matter injury on MRI. The red nucleus of the midbrain was the only gray matter structure damaged on MR imaging [41, 42].

A lack of injury to the basal ganglia nuclei in these models closely aligns with the early premature neonate after HI injury. Human neonates at these premature age extremes can be expected to experience damage in the brainstem which may include the red nucleus of the midbrain seen in the rabbit model. Additionally, injury to the corpus callosum, anterior commissure, and corona radiata white tracts can be expected to occur in preterm neonates. Due to the paucity of literature utilizing rabbit models that mimic HIE in the preterm neonate, the ability to translate this model into human preterm neonatal MRI is still unclear. Additionally, the limited follow-up imaging of the rabbit models should also be further investigated to determine long-term brain imaging outcomes.

#### 3.2.3 Non-Human Primate Model

The *Macaca nemestrina* (pig-tailed macaque) has also been utilized as a model of HIE. The age of the macaque used for HIE has been between 164 to 173 days of gestation with full gestation being 173 days. The brain development of the animals at this level of maturity are comparable to a late preterm infant [43]. Injury is induced through umbilical cord occlusion for 12 to 18 minutes immediately prior to delivery. One study also emulated episodes of apnea by exposing the newborn to 8% oxygen for 3 minutes up to 8 times after the animal was delivered [43]. MRI and MRS have been performed between day 1 and day 8 for initial analysis of brain injury, with repeat imaging performed at 2 months, 6 months, or 9 months of age.

MR imaging demonstrated T2-weighted hyper-intense regions within the bilateral Ventral Posterolateral (VPL) thalami and putamen in one study [43]. However, MRI studies largely did not demonstrate significant differences between the experimental and control models near birth or at long-term repeat imaging. DWI was found to be normal for all animals and there were no changes demonstrated in total brain volume, ischemic changes, or cyst formation near birth or at two months after injury. MR spectroscopy within the first 8 days of life did demonstrate increased choline and lactate, elevated myoinositol peak, decreased N-Acetylaspartate (NAA), and decreased NAA/Creatine ratio [43-45].

Non-human primates provided the closest model for mimicking HIE in the neonate due to their physiological and anatomical similarities. Strengths of the model include similar areas of injury within the thalamic nuclei as well as consistent abnormalities on MRS. The lack of significant injury at long-term follow-up, however, is not consistent with human preterm brain injury. Although this model provides a close equivalent representation of the human neonate, it is limited by the significantly increased cost of studies using macaque over small animal models.

#### 3.2.4 Sheep Model

Two strains of sheep have been commonly used to model neonatal HIE: Western Breed Sheep and Romney-Suffolk Cross Sheep. The gestational age at time of injury in published studies ranges between 88 to 97 days gestation, which is approximately equivalent to a 24 to 28 week preterm neonate [46-48]. Bilateral CCAO is applied for 30 to 37 minutes to induce HI injury, and MRI has been performed at day 3, 7, and 14 after injury.

Imaging on day 3 with T1- and T2-weighted MRI demonstrated cerebral hemisphere irregularity or loss of the cortical ribbon (a thin layer of gray matter around the cerebral hemispheres) and indistinct cortical signal. Additionally, the subcortical area and PVWM displayed diffuse increased T2 signal and decreased T1 signal [46]. T2 MRI on day 7 demonstrated diffuse hyperintense areas in the deep and sub-gyral white matter along with focal hyperintensity in the superficial gyri. Lastly, imaging on day 14 with T2 MRI was found to display diffuse hypointensity in the deep and sub-gyral white matter, focal hyperintensity in the superficial gyri, and focal hyperintensity within the PVWM [49].

Overall, these sheep with a human brain equivalency of 24 – 28 weeks gestation demonstrated damage to areas that are grossly in accordance with the injury observed in preterm neonates after HI injury, such as the PVWM, and deep white matter of the subcortical areas. Injury to gray matter in the cortex would not be expected in the preterm neonates. One of the largest gaps in this literature lies with the limited range of maturity that has been investigated in the sheep model. Additional research utilizing a model with maturity similar to the late preterm infant would allow for an assessment of whether the injury patterns mature in this model in the same manner as the human preterm infants.

### 3.3 Magnetic Resonance Imaging of Term Neonates

MRI findings in term neonates after HIE vary depending on the severity and the timing after insult. Severe HIE is characterized on MRI by a central pattern injury that includes the thalamus, PLIC, and hippocampus with the most severe HIE resulting in involvement of the entire cortex [50, 51]. Imaging with T1-weighted MRI within the first two to three days following insult will typically show subtle or no signal change, however imaging at days three to seven will show mild increased signal abnormality in the posterolateral putamen and bilateral VL thalami. One may also see preservation of the hyperintense signal in the PLIC [50, 52]. Contrast these findings with what is seen with moderate HIE injury where there is a further extension of the T1-weighted hyperintensity more towards the anterior portions of the putamen and posteromedially in the thalamus. With moderate HIE, abnormal signal in the PLIC is not as consistent as with severe injury [50, 52]. When PLIC abnormalities are present, it is typical to see a steady increase in size over the first week. A focal hyperintensity on T1-weighted imaging is seen in the posterior putamina, VL thalami, and periolandic cortex in the second week after injury [52]. After the second week, T1 signal intensity can be present for several months [53].

On T2-weighted imaging in term neonates there also tends be minimal signal changes in the first days following injury, although some studies have shown isointensity of the posterior thalami secondary to vasogenic edema [17, 54]. With more severe injury injury, T2 imaging may demonstrate more damage within the bilateral thalami, lentiform nuclei, and head of the caudate lobe with loss of hypointensity of the PLIC. With T2 imaging in the second week, increased signal intensity is seen in the basal ganglia and thalamus [14, 53, 55]. This increased intensity in T2 can be seen for several months, and if injury progresses to the chronic stage, MRI generally demonstrates cerebral atrophy.

As opposed to T1 and T2-weighted imaging, DWI shows decreased diffusion in the VL thalami within the first 24 hours after injury. It is important to note that DWI will still underestimate the extent of HI injury if imaging is performed early, it will appropriately reflect injury up until the fifth day, and then will pseudonormalize near the eighth to tenth day [56, 57]. MR spectroscopy allows for detection of lactate when performed in the first week after injury, with levels decreasing after the first week, but still detectable up to several months of life. Levels of NAA, which is a measure of brain integrity, allow for utilizing a ratio of lactate NAA as a marker of the severity of HIE in the subacute and chronic stages of injury [58].

#### 3.3.1 Murine Model

Murine models used to mimic HIE in a term neonate have utilized the same strains of animals as the studies modeling preterm HIE, however the animals are older, at postnatal day 9 to 12 [3, 59-62]. Methods used for inducing HI injury in these animals were either unilateral CCAO, bilateral CCAO, or MCAO. Exposure to 8% oxygen was performed between 1 and 65 minutes. MRI was performed at 24 hours, 14 days, 18 days, 21 days, 25 days, 28 days, or 30 days after HI injury.

MRI findings in the term murine model after moderate HI injury are likely to display cortical damage with additional damage to the striatum. As severity of injury increases, the affected areas of the murine brain can be expected to also include the thalamus, basal ganglia, corpus callosum and hippocampus [59-63]. The hippocampal atrophy seen with MR imaging has been further defined through pathological analysis to be specific to the CA1 layer [59]. Pathological analysis has also narrowed the thalamic injury to the ventroposterior thalamic nucleus [60].

The thalamic, hippocampal, and basal ganglia, including the striatum, injury seen in the term murine model is similar to what has been seen in imaging of term human neonates. Damage to the corpus callosum seen in this model, however, is not commonly demonstrated in equivalent human imaging. It is worth noting that significant research has been performed utilizing the postnatal day 7 murine to model term neonatal HIE. This model, however, is more equivalent to late preterm neonates at 33 to 34 weeks gestation, so studies assessing term injury should consider utilizing animals between postnatal days 9 and 12.

#### 3.3.2 Rabbit Model

New Zealand White Rabbits between 4-14 days postnatal days of age have also been used to mimic HIE in the term neonate. Animals at this age are equivalent to a term neonate at 40 weeks gestation [1]. Unilateral CCAO is utilized with 10% oxygen exposure to induce HI effect. One study allowed HI injury to occur between 1 and 2.5 hours while performing DWI and perfusion imaging every 15 to 30 minutes during HI injury induction [64]. Another study performed CCAO ligation with 10% oxygen exposure while continuous MRI was performed. These animals were then sacrificed and underwent brain imaging for 5 more minutes [65].

Initial MRI findings of rabbits after undergoing HI injury demonstrated focal diffusion changes within the ipsilateral cortex, hippocampus, and thalamus. As injury continued to occur, ischemic changes then developed in the ipsilateral subcortical white matter before developing in the contralateral superficial cerebral cortex. Further continuous imaging finally showed progression into the contralateral subcortical white matter and basal ganglia. Small areas of the brain including the caudate and putamen were not clearly delineated during imaging [64, 65].

These studies are not easily translatable to human neonates because imaging is not generally performed during the hypoxic injury. Attempting to compare the areas of injury, however, there do appear to be immediate similarity to term human infants, with involvement of the thalamus, hippocampus, cortical, and subcortical structures including striatum, PLIC, and basal ganglia. Future research utilizing the rabbit model for modeling HIE in term neonates will be beneficial due to the paucity of studies. Because the imaging in the previous rabbit studies occurred during and shortly after HI injury, future research imaging at a similar time frame to human infants would allow for better clinical translation.

#### 3.3.3 Piglet Model

Piglets used in modeling term HIE range between 115 days gestation and postnatal day 7, with full gestation in piglets being equivalent to a 36 to 38 week human neonate [3, 66, 67]. HI injury is induced *via* bilateral CCAO with between 4% and 12% oxygen exposure [68]. The range of low oxygen exposure has been wide between 5 and 91 minutes. MRI and MRS analyses were performed up to 7 days after HI injury [67-71].

In two studies, MRI demonstrated decreased ADC in the basal ganglia and increased T2-weighted signaling in the striatum [67, 71]. Another study, however, found no basal ganglia injury, but increased T2 signaling was visualized in the subcortical parietal white matter 3 days after injury [70]. These studies are not easily comparable, due to the use of different end points for termination of hypoxia. One study continued hypoxia for up to 60 minutes as long as the piglet remained clinically stable, while others used target levels of phosphocreatine/inorganic phosphate signal on MRS. Results of MRS demonstrated elevated lactate, lactate/NAA, lactate/creatine, and lactate/choline up to 7 days after HI injury [67, 68, 70]. Other measured compounds not found to be statistically significant after injury included inorganic phosphate, phosphocreatine, adenosine triphosphate, phosphomonoester, phosphodiester, and intracellular pH [69].

MRI findings of the piglet model displayed inconsistent findings of brain damage after HI injury in the basal ganglia and subcortical white matter, however this may be due to differences in injury endpoints [67, 70, 71]. In the future, it will be important for better standardization of the piglet model when inducing sufficient HI injury. The wide range of HI injury duration (5 to 91 minutes) and utilization of MRS findings rather than standardized time to determine HI injury may attribute to the varied MRI findings. The MR spectroscopy findings seen in the piglet model align with expected MR spectroscopy findings that can be seen in the term neonate after HI injury where elevated lactate and lactate/NAA has been described.

## CONCLUSION

Multiple animal models have been utilized to mimic the effects of HIE in the neonate with varying degrees of replication accuracy. The majority of animal studies have assessed neuropathological endpoints, however the translatability to humans is unclear due to the paucity of human pathological data in the era of therapeutic hypothermia. Further MRI-based studies with these established animal models would potentially allow for better comparison between these models and human neonates due to the well-defined MR imaging findings in humans. Non-human primate models provide the closest imaging correlate to humans, however the expense and ethical concerns with large animal research often restricts their use. As other animal models of HIE continue to evolve it will be important for investigators to include a focus on comparing imaging findings to human studies, and not just pathologic findings. A better understanding of the similarities and differences between these animal models and humans will ultimately aid in designing future effective translational research on diagnostic studies and therapies for the treatment of HIE.

## LIST OF ABBREVIATIONS

HIE: = Hypoxic-Ischemic Encephalopathy
MRI: = Magnetic Resonance Imaging
HI: = Hypoxic-Ischemic
MR: = Magnetic Resonance
DWI: = Diffusion-Weighted Imaging
ADC: = apparent Diffusion Coefficient
MRS: = Magnetic Resonance Spectroscopy
PLIC: = Posterior Limb of Internal Capsule
CCAO: = Common Carotid Artery Occlusion
MCAO: = Middle Cerebral Artery Occlusion
PVWM: = Periventricular White Matter
VL: = Ventrolateral
VPL: = Ventral Posterolateral
NAA: = N-Acetylaspartate

## References

[1] Workman A.D., Charvet C.J., Clancy B., Darlington R.B., Finlay B.L. (2013). Modeling transformations of neurodevelopmental sequences across mammalian species.. J. Neurosci..

[2] Verheul H.B., Berkelbach van der Sprenkel J.W., Tulleken C.A., Tamminga K.S., Nicolay K. (1992). Temporal evolution of focal cerebral ischemia in the rat assessed by T2-weighted and diffusion-weighted magnetic resonance imaging.. Brain Topogr..

[3] Huang L., Zhao F., Qu Y., Zhang L., Wang Y., Mu D. (2017). Animal models of hypoxic-ischemic encephalopathy: Optimal choices for the best outcomes.. Rev. Neurosci..

[4] Miyabe M., Mori S., van Zijl P.C., Kirsch J.R., Eleff S.M., Koehler R.C., Traystman R.J. (1996). Correlation of the average water diffusion constant with cerebral blood flow and ischemic damage after transient middle cerebral artery occlusion in cats.. J. Cereb. Blood Flow Metab..

[5] van Bruggen N., Cullen B.M., King M.D., Doran M., Williams S.R., Gadian D.G., Cremer J.E. (1992). T2- and diffusion-weighted magnetic resonance imaging of a focal ischemic lesion in rat brain.. Stroke.

[6] Zimmer R., Lang R., Oberdörster G. (1971). Post-ischemic reactive hyperemia of the isolated perfused brain of the dog.. Pflugers Arch..

[7] Busza A.L., Allen K.L., King M.D., van Bruggen N., Williams S.R., Gadian D.G. (1992). Diffusion-weighted imaging studies of cerebral ischemia in gerbils. Potential relevance to energy failure.. Stroke.

[8] Moseley M.E., Cohen Y., Mintorovitch J., Chileuitt L., Shimizu H., Kucharczyk J., Wendland M.F., Weinstein P.R. (1990). Early detection of regional cerebral ischemia in cats: Comparison of diffusion- and T2-weighted MRI and spectroscopy.. Magn. Reson. Med..

[9] van Dorsten F.A., Hata R., Maeda K., Franke C., Eis M., Hossmann K.A., Hoehn M. (1999). Diffusion- and perfusion-weighted MR imaging of transient focal cerebral ischaemia in mice.. NMR Biomed..

[10] Hesselbarth D., Franke C., Hata R., Brinker G., Hoehn-Berlage M. (1998). High resolution MRI and MRS: A feasibility study for the investigation of focal cerebral ischemia in mice.. NMR Biomed..

[11] Preston D.

[12] Hoyer C., Gass N., Weber-Fahr W., Sartorius A. (2014). Advantages and challenges of small animal magnetic resonance imaging as a translational tool.. Neuropsychobiology.

[13] Logitharajah P., Rutherford M.A., Cowan F.M. (2009). Hypoxic-ischemic encephalopathy in preterm infants: Antecedent factors, brain imaging, and outcome.. Pediatr. Res..

[14] Barkovich A.J., Sargent S.K. (1995). Profound asphyxia in the premature infant: Imaging findings.. Am. J. Neuroradiol..

[15] Zhang F., Liu C., Qian L., Hou H., Guo Z. (2016). Diffusion tensor imaging of white matter injury caused by prematurity-induced hypoxic-ischemic brain damage.. Med. Sci. Monit..

[16] Varghese B., Xavier R., Manoj V.C., Aneesh M.K., Priya P.S., Kumar A., Sreenivasan V.K. (2016). Magnetic resonance imaging spectrum of perinatal hypoxic-ischemic brain injury.. Indian J. Radiol. Imaging.

[17] Barkovich A.J. (2005). Pediatric neuroimaging..

[18] Robertson N.J., Lewis R.H., Cowan F.M., Allsop J.M., Counsell S.J., Edwards A.D., Cox I.J. (2001). Early increases in brain myo-inositol measured by proton magnetic resonance spectroscopy in term infants with neonatal encephalopathy.. Pediatr. Res..

[19] Malik G.K., Pandey M., Kumar R., Chawla S., Rathi B., Gupta R.K. (2002). MR imaging and *in vivo* proton spectroscopy of the brain in neonates with hypoxic ischemic encephalopathy.. Eur. J. Radiol..

[20] Robertson N.J., Kuint J., Counsell T.J., Rutherford T.A., Coutts A., Cox I.J., Edwards A.D. (2000). Characterization of cerebral white matter damage in preterm infants using 1H and 31P magnetic resonance spectroscopy.. J. Cereb. Blood Flow Metab..

[21] Inder T.E., Warfield S.K., Wang H., Hüppi P.S., Volpe J.J. (2005). Abnormal cerebral structure is present at term in premature infants.. Pediatrics.

[22] Ment L.R., Vohr B., Allan W., Katz K.H., Schneider K.C., Westerveld M., Duncan C.C., Makuch R.W. (2003). Change in cognitive function over time in very low-birth-weight infants.. JAMA.

[23] Nosarti C., Giouroukou E., Healy E., Rifkin L., Walshe M., Reichenberg A., Chitnis X., Williams S.C., Murray R.M. (2008). Grey and white matter distribution in very preterm adolescents mediates neurodevelopmental outcome.. Brain.

[24] Dyet L.E., Kennea N., Counsell S.J., Maalouf E.F., Ajayi-Obe M., Duggan P.J., Harrison M., Allsop J.M., Hajnal J., Herlihy A.H., Edwards B., Laroche S., Cowan F.M., Rutherford M.A., Edwards A.D. (2006). Natural history of brain lesions in extremely preterm infants studied with serial magnetic resonance imaging from birth and neurodevelopmental assessment.. Pediatrics.

[25] Shoykhet M., Simons D.J., Alexander H., Hosler C., Kochanek P.M., Clark R.S. (2012). Thalamocortical dysfunction and thalamic injury after asphyxial cardiac arrest in developing rats.. J. Neurosci..

[26] Wilson M.D. (2015). Animal models of cerebral palsy: Hypoxic brain injury in the newborn.. Iran. J. Child. Neurol..

[27] Jelinski S.E., Yager J.Y., Juurlink B.H. (1999). Preferential injury of oligodendroblasts by a short hypoxic-ischemic insult.. Brain Res..

[28] Rice J.E., Vannucci R.C., Brierley J.B. (1981). The influence of immaturity on hypoxic-ischemic brain damage in the rat.. Ann. Neurol..

[29] Schwartz P.H., Massarweh W.F., Vinters H.V., Wasterlain C.G. (1992). A rat model of severe neonatal hypoxic-ischemic brain injury.. Stroke.

[30] Uehara H., Yoshioka H., Kawase S., Nagai H., Ohmae T., Hasegawa K., Sawada T. (1999). A new model of white matter injury in neonatal rats with bilateral carotid artery occlusion.. Brain Res..

[31] Baud O., Daire J.L., Dalmaz Y., Fontaine R.H., Krueger R.C., Sebag G., Evrard P., Gressens P., Verney C. (2004). Gestational hypoxia induces white matter damage in neonatal rats: A new model of periventricular leukomalacia.. Brain Pathol..

[32] Aggarwal M., Burnsed J., Martin L.J., Northington F.J., Zhang J. (2014). Imaging neurodegeneration in the mouse hippocampus after neonatal hypoxia-ischemia using oscillating gradient diffusion MRI.. Magn. Reson. Med..

[33] Huang H.Z., Wen X.H., Liu H. (2016). Sex differences in brain MRI abnormalities and neurodevelopmental outcomes in a rat model of neonatal hypoxia-ischemia.. Int. J. Neurosci..

[34] Qiao M., Meng S., Scobie K., Foniok T., Tuor U.I. (2004). Magnetic resonance imaging of differential gray *versus* white matter injury following a mild or moderate hypoxic-ischemic insult in neonatal rats.. Neurosci. Lett..

[35] Adén U., Dahlberg V., Fredholm B.B., Lai L.J., Chen Z., Bjelke B. (2002). MRI evaluation and functional assessment of brain injury after hypoxic ischemia in neonatal mice.. Stroke.

[36] Albensi B.C., Schweizer M.P., Rarick T.M., Filloux F. (1998). Magnetic resonance imaging of hypoxic-ischemic brain injury in the neonatal rat.. Invest. Radiol..

[37] Wang S., Wu E.X., Tam C.N., Lau H.F., Cheung P.T., Khong P.L. (2008). Characterization of white matter injury in a hypoxic-ischemic neonatal rat model by diffusion tensor MRI.. Stroke.

[38] Ten V.S., Wu E.X., Tang H., Bradley-Moore M., Fedarau M.V., Ratner V.I., Stark R.I., Gingrich J.A., Pinsky D.J. (2004). Late measures of brain injury after neonatal hypoxia-ischemia in mice.. Stroke.

[39] Wang Y., Cheung P.T., Shen G.X., Wu E.X., Cao G., Bart I., Wong W.H., Khong P.L. (2006). Hypoxic-ischemic brain injury in the neonatal rat model: Relationship between lesion size at early MR imaging and irreversible infarction.. AJNR Am. J. Neuroradiol..

[40] Buser J.R., Segovia K.N., Dean J.M., Nelson K., Beardsley D., Gong X., Luo N.L., Ren J., Wan Y., Riddle A., McClure M.M., Ji X., Derrick M., Hohimer A.R., Back S.A., Tan S. (2010). Timing of appearance of late oligodendrocyte progenitors coincides with enhanced susceptibility of preterm rabbit cerebral white matter to hypoxia-ischemia.. J. Cereb. Blood Flow Metab..

[41] Derrick M., Drobyshevsky A., Ji X., Tan S. (2007). A model of cerebral palsy from fetal hypoxia-ischemia.. Stroke.

[42] Drobyshevsky A., Derrick M., Wyrwicz A.M., Ji X., Englof I., Ullman L.M., Zelaya M.E., Northington F.J., Tan S. (2007). White matter injury correlates with hypertonia in an animal model of cerebral palsy.. J. Cereb. Blood Flow Metab..

[43] McAdams R.M., McPherson R.J., Kapur R.P., Juul S.E. (2017). Focal brain injury associated with a model of severe hypoxic-ischemic encephalopathy in nonhuman primates.. Dev. Neurosci..

[44] Jacobson Misbe E.N., Richards T.L., McPherson R.J., Burbacher T.M., Juul S.E. (2011). Perinatal asphyxia in a nonhuman primate model.. Dev. Neurosci..

[45] Juul S.E., Aylward E., Richards T., McPherson R.J., Kuratani J., Burbacher T.M. (2007). Prenatal cord clamping in newborn Macaca nemestrina: A model of perinatal asphyxia.. Dev. Neurosci..

[46] Fraser M., Bennet L., Helliwell R., Wells S., Williams C., Gluckman P., Gunn A.J., Inder T. (2007). Regional specificity of magnetic resonance imaging and histopathology following cerebral ischemia in preterm fetal sheep.. Reprod. Sci..

[47] Barlow R.M. (1969). The foetal sheep: morphogenesis of the nervous system and histochemical aspects of myelination.. J. Comp. Neurol..

[48] McIntosh G.H., Baghurst K.I., Potter B.J., Hetzel B.S. (1979). Foetal brain development in the sheep.. Neuropathol. Appl. Neurobiol..

[49] Riddle A., Dean J., Buser J.R., Gong X., Maire J., Chen K., Ahmad T., Cai V., Nguyen T., Kroenke C.D., Hohimer A.R., Back S.A. (2011). Histopathological correlates of magnetic resonance imaging-defined chronic perinatal white matter injury.. Ann. Neurol..

[50] Okereafor A., Allsop J., Counsell S.J., Fitzpatrick J., Azzopardi D., Rutherford M.A., Cowan F.M. (2008). Patterns of brain injury in neonates exposed to perinatal sentinel events.. Pediatrics.

[51] Shroff M.M., Soares-Fernandes J.P., Whyte H., Raybaud C. (2010). MR imaging for diagnostic evaluation of encephalopathy in the newborn.. Radiographics.

[52] Mercuri E., Ricci D., Cowan F.M., Lessing D., Frisone M.F., Haataja L., Counsell S.J., Dubowitz L.M., Rutherford M.A. (2000). Head growth in infants with hypoxic-ischemic encephalopathy: Correlation with neonatal magnetic resonance imaging.. Pediatrics.

[53] Barkovich A.J.M.R. (1992). MR and CT evaluation of profound neonatal and infantile asphyxia.. AJNR Am. J. Neuroradiol..

[54] Hagmann CF, Rennie JM, Robertson NJ (2008). Neonatal cerebral investigation..

[55] Huang B.Y., Castillo M. (2008). Hypoxic-ischemic brain injury: imaging findings from birth to adulthood. Radiographics: A review publication of the Radiological Society of North America.. Inc.

[56] Rutherford M., Srinivasan L., Dyet L., Ward P., Allsop J., Counsell S., Cowan F. (2006). Magnetic resonance imaging in perinatal brain injury: Clinical presentation, lesions and outcome.. Pediatr. Radiol..

[57] Barkovich A.J. (2006). MR imaging of the neonatal brain.. Neuroimaging Clin. N. Am..

[58] Roelants-Van Rijn A.M., van der Grond J., de Vries L.S., Groenendaal F. (2001). Value of (1) H-MRS using different echo times in neonates with cerebral hypoxia-ischemia.. Pediatr. Res..

[59] Recker R., Adami A., Tone B., Tian H.R., Lalas S., Hartman R.E., Obenaus A., Ashwal S. (2009). Rodent neonatal bilateral carotid artery occlusion with hypoxia mimics human hypoxic-ischemic injury.. J. Cereb. Blood Flow Metab..

[60] Tsuji M., Ohshima M., Taguchi A., Kasahara Y., Ikeda T., Matsuyama T. (2013). A novel reproducible model of neonatal stroke in mice: Comparison with a hypoxia-ischemia model.. Exp. Neurol..

[61] Patel S.D., Pierce L., Ciardiello A., Hutton A., Paskewitz S., Aronowitz E., Voss H.U., Moore H., Vannucci S.J. (2015). Therapeutic hypothermia and hypoxia-ischemia in the term-equivalent neonatal rat: Characterization of a translational preclinical model.. Pediatr. Res..

[62] Burnsed J.C., Chavez-Valdez R., Hossain M.S., Kesavan K., Martin L.J., Zhang J., Northington F.J. (2015). Hypoxia-ischemia and therapeutic hypothermia in the neonatal mouse brain--a longitudinal study.. PLoS One.

[63] Dzietko M., Wendland M., Derugin N., Ferriero D.M., Vexler Z.S. (2011). Magnetic Resonance Imaging (MRI) as a translational tool for the study of neonatal stroke.. J. Child Neurol..

[64] D’Arceuil H.E., de Crespigny A.J., Röther J., Seri S., Moseley M.E., Stevenson D.K., Rhine W. (1998). Diffusion and perfusion magnetic resonance imaging of the evolution of hypoxic ischemic encephalopathy in the neonatal rabbit.. J. Magn. Reson. Imaging.

[65] D’Arceuil H.E., Crespigny A.J., Röther J., Moseley M., Rhine W. (1999). Serial magnetic resonance diffusion and hemodynamic imaging in a neonatal rabbit model of hypoxic-ischemic encephalopathy.. NMR Biomed..

[66] Dickerson J.W., Dobbing J. (1967). Prenatal and postnatal growth and development of the central nervous system of the pig.. Proc. R. Soc. Lond. B Biol. Sci..

[67] Munkeby B.H., De Lange C., Emblem K.E., Bjørnerud A., Kro G.A., Andresen J., Winther-Larssen E.H., Løberg E.M., Hald J.K. (2008). A piglet model for detection of hypoxic-ischemic brain injury with magnetic resonance imaging.. Acta Radiol..

[68] Penrice J., Lorek A., Cady E.B., Amess P.N., Wylezinska M., Cooper C.E., D’Souza P., Brown G.C., Kirkbride V., Edwards A.D., Wyatt J.S., Reynolds E.O. (1997). Proton magnetic resonance spectroscopy of the brain during acute hypoxia-ischemia and delayed cerebral energy failure in the newborn piglet.. Pediatr. Res..

[69] McCulloch K.M., Raju T.N., Navale S., Burt C.T., Roohey T., Moustogiannis A., Zachary J.F. (2005). Developing a long-term surviving piglet model of neonatal hypoxic-ischemic encephalopathy.. Neurol. Res..

[70] Vial F., Serriere S., Barantin L., Montharu J., Nadal-Desbarats L., Pourcelot L., Seguin F. (2004). A newborn piglet study of moderate hypoxic-ischemic brain injury by 1H-MRS and MRI.. Magn. Reson. Imaging.

[71] Gang Q., Zhang J., Hao P., Xu Y. (2013). Detection of hypoxic-ischemic brain injury with 3D-enhanced T2* weighted angiography (ESWAN) imaging.. Eur. J. Radiol..

